# Knowledge, attitude, and practice assessment toward COVID-19 among communities in East Nusa Tenggara, Indonesia: A cross-sectional study

**DOI:** 10.3389/fpubh.2022.957630

**Published:** 2022-10-31

**Authors:** Felix Lee, Aileen Alessandra Suryohusodo

**Affiliations:** ^1^Faculty of Medicine, University of Indonesia, Depok, Indonesia; ^2^Faculty of Medicine, Atma Jaya Catholic University of Indonesia, Jakarta, Indonesia

**Keywords:** attitude, correlates, COVID-19, Indonesia, knowledge, practice

## Abstract

**Objective:**

This research aimed to assess the knowledge, attitude and practice toward COVID-19 among East Nusa Tenggara (NTT) population and identify associated sociodemographic factors.

**Methods:**

A cross-sectional study was performed among communities in 22 regencies of NTT between October–November 2021 using a structured and validated questionnaire that consists of 22-items of knowledge, 6-items of attitude and 13-items of practice related COVID-19 questions. A cut off point of at least 70% was employed to categorize good knowledge, attitude, and practice.

**Results:**

The percentages of survey respondents showing good knowledge, attitude and practice toward COVID-19 were 79.8, 72.7, and 94.6%, respectively. There was a significant positive, though weak, linear correlation between knowledge and practice scores (ρ = 0.097; *p* = 0.049). Knowledge was strongly associated with regency of residence (Cramer's V = 0.266; *p* = 0.010), education (Cramer's V = 0.312; *p* < 0.001), and occupation (Cramer's V = 0.313; *p* < 0.001). Attitude and practice had strong relationship with regency of residence (Cramer's V = 0.289; *p* = 0.024) and education (Cramer's V = 0.272; *p* < 0.001), respectively.

**Conclusion:**

Nearly all survey participants showed good precautionary behaviors, but there was still a quarter of respondents indicated poor knowledge and attitude. There was also a positive relationship between knowledge and practice scores thereby indicating the importance of local public health advocates to distribute information uniformly especially to the groups with inadequate knowledge toward COVID-19 as a means to control the virus transmission.

## Introduction

Coronavirus disease 2019 (COVID-19) is caused by severe acute respiratory syndrome coronavirus 2 (SARS-CoV-2). People who contract COVID-19 mainly experience fever, dry cough, and fatigue. The first reported COVID-19 case was in Wuhan, China, in December 2019. It has henceforth spread rapidly to other countries which led to a global pandemic (1). Indonesian officials confirmed its first case in the country on March 2, 2020, and subsequently led to uncontrollable surge of COVID-19 cases across the country. From July to August 2021, Indonesia was hit with COVID-19 second wave with daily new cases peaking at almost 57,000 which was the highest globally at that time. Moreover, all health facilities were incapable of meeting the increasing demand and hence the government had enforced a strict lockdown across the country (2).

East Nusa Tenggara (NTT) is the southernmost province of Indonesia that constitutes the easter part of the Lesser Sunda Islands. It consists of 22 administrative regencies which spread among 500 islands (3). During the COVID-19 second wave in Indonesia, NTT accounted for one of the highest daily confirmed cases reported in Indonesia with a peak of 3,598 new cases in one day, despite having population of two percent of the total Indonesian population. Mortality rate in NTT due to COVID-19 increased by 68% and there was a week where more than half of all tested individuals was confirmed positive with the virus (2).

Knowledge, attitude, and practice (KAP) assessment is often conducted to identify knowledge gaps and behavioral patterns in a particular population with the aim to implement and strengthen effective public health interventions. Understanding KAP in a population and its sociodemographic factors have been shown to play a beneficial role in handling outbreaks. For example, one study found that influenza had a higher burden on older and impoverished population. Hence, urging public health authorities to divert more attention toward these groups (4).

Public knowledge and attitude toward COVID-19 are likely to influence their adherence to good COVID-19 practices which are essential to slow down the virus transmission. Therefore, it is important to know the existing KAP of NTT residents on COVID-19 and also determine the factors influencing the public to adopt appropriate practices and attitudes in order for local health policy makers to create more effective strategies with the aim to suppress the alarming rise of COVID-19 cases (5, 6). To the date when this study was performed, there was no research done describing the KAP of general NTT population toward COVID-19 comprehensively. There was one study conducted which assessed KAP of all Indonesian population on COVID-19. However, it only included one participant from NTT which was far from sufficient to represent the province that inhabited by more than five million people (7). Unlike previous similar studies in other countries and regions, the questionnaire used in this study covered a more extensive items and was updated based on the evolving information in regards with COVID-19, therefore providing a much more reliable description on the current KAP toward the outbreak (8–10). Our study also included extensive statistical analysis, which strengthens the description of association. Hence, this study aimed to evaluate KAP of COVID-19 and the possible sociodemographic factors associated with it among NTT communities. From this study, the local public health planners could potentially formulate more effective and personalized strategies to control the COVID-19 pandemic in the region.

## Methods

A cross-sectional study was conducted among population living in the NTT province of Indonesia from October 26 to November 7, 2021. To recruit participants, a convenience sampling method was performed. Sample size requirement was calculated with the formula: $\frac{{Z_{1-\propto /2}}^{2}p\left(1-p\right)}{d^{2}}$, where the standard normal variate (*Z*_1−∝/2_) was 1.96 with 0.05 as the chosen α (95% confidence), the expected proportion population (p) being 0.5, and margin of error (d) was 5% (11). Hence, a minimum of 384 respondents were needed to reach adequate power for this study. Inclusion criteria included: (i) individuals aged 18 years and older, (ii) permanent resident of NTT, and (iii) agreeing or signing consent form to fill out survey and be included in this study. Exclusion criteria included: (i) illiterate individuals, (ii) unable to understand Indonesian language, and (iii) incomplete questionnaire. To represent the whole province, this study covered all 22 regencies on more than 12 islands across NTT which is part of the Lesser Sunda Islands archipelago.

### Survey instrument

A structured questionnaire encompassing socio-demographic information, previous COVID-19 infection, comorbidities and COVID-19 KAP was designed, validated and then administered. Socio-demographic information consisted of respondent's age sex, education, occupation and regency of residence. Based on similar published studies on COVID-19 (12–15), 22-items of knowledge, 6-items of attitude and 13-items of practice related COVID-19 questions were developed.

### Data collection

The questionnaire was first written in Indonesian language and had been field-tested for comprehension and suitability across the target population. It was then duplicated in the form of paper questionnaires and online survey *via* Google Form (Google LLC, California, USA). The paper questionnaires were distributed randomly by data collectors to every neighborhood and village through the aid of local community health centers and villages' head. Verbal and written inform consent were taken from all respondents. The online form was shared with a link through social media platform and forwarded by colleagues to individuals particularly living in the remote areas or hardly accessible by data collectors. All participants were required to electronically agree and give consent in completing the survey for research purpose before they can access and submit the questionnaire online. Collection of data, either through online or paper-based, was carried out in all 22 regencies of NTT province. This study obtained ethical approval from Faculty of Public Health Universitas Airlangga – ERB Registration No. 47/EA/KEPK/2021.

### Measures

Twenty-two items in the knowledge section were equally weighted, dichotomized, and a total score was generated by the sum of correct answers with maximum of 22. A score of 16 and above was categorized as having good COVID-19 knowledge, otherwise classified as bad knowledge. The same applied to attitude section, but with a maximum score of six and a minimum of five was required to be classified as good attitude. Good attitude was interpreted as being positive, taking COVID-19 seriously and aware of the importance in following public health measures to reduce the impact of the pandemic.

In the practice section, all items were uniformly weighted and had four rating scales in which never, rarely, occasionally and always scored as 1, 2, 3, and 4, respectively. However, two out of the 13 items had reverse scoring to minimize acquiescence bias. Hence, the maximum score that could be reached was 52 with 37 as the minimum score to be categorized as good practice or behavior. The items in knowledge, attitude and practice were clearly shown in Tables 1, 2.

**Table 1:** Frequencies and percentages of every response in each item in knowledge and attitude section.

Item^*^	**True (*n, %*)**	**False (*n, %*)**
**Knowledge section**		
COVID-19 is a disease caused by coronavirus	391 (95.37%)	19 (4.63%)
COVID-19 is a disease that is not dangerous and is similar with influenza	38 (9.27%)	372 (90.73%)
Main symptoms of COVID-19 are fever, fatigue, cough, and myalgia	357 (87.07%)	53 (12.93%)
People with COVID-19 that show no signs are called asymptomatic patients	371 (90.49%)	39 (9.51%)
COVID-19 symptoms are usually worse in elderly compared to younger people	364 (88.78%)	46 (11.21%)
COVID-19 patients with chronic diseases, such as diabetes (high blood glucose), cardiovascular disease, and obesity usually experience more severe condition	375 (91.46%)	35 (8.53%)
Children and teenagers do not need to take any precautions in preventing the spread of COVID-19, since they have a stronger immune system	79 (19.27%)	331 (80.73%)
People who have a strong immune system cannot be infected with COVID-19	234 (57.07%)	176 (42.93%)
People infected with COVID-19 but show no symptoms cannot spread the virus to other people	81 (19.76%)	329 (80.24%)
Coronavirus can spread through respiratory droplets from an infected person	385 (93.90%)	25 (6.10%)
COVID-19 infection could be transmitted through food and contact with wild animals	143 (34.88%)	267 (65.12%)
Corpses of COVID-19 patients that have not been buried can be a source for the spread of the virus	354 (86.34%)	56 (13.66%)
Corpses of COVID-19 patients that have been buried can be a source for the virus transmission	86 (20.98%)	324 (79.02%)
Masks that are made from fabric cannot be penetrated by the coronavirus	181 (44.15%)	229 (55.85%)
COVID-19 only transmits through objects and not through air	150 (36.59%)	260 (63.41%)
There is no proven effective drug for COVID-19 to date, but early symptomatic and supportive treatment can help most COVID-19 patients to recover	365 (89.02%)	45 (10.98%)
To prevent the transmission of COVID-19 infection, people must avoid traveling to crowded places and avoid using public transportation	384 (93.66%)	26 (6.34%)
The transmission of COVID-19 can be prevented by not touching the face	326 (79.51%)	84 (20.49%)
Healthy people do not need to wear masks when leaving the house	41 (10.00%)	369 (90.00%)
Coronavirus can survive several hours outside the human body	335 (81.71%)	75 (18.29%)
Self-isolation is not necessary for asymptomatic COVID-19 patients	129 (31.46%)	281 (68.54%)
Self-isolation and prompt treatment for people infected with COVID-19 are the most effective way to reduce the spread of the virus	398 (97.07%)	12 (2.93%)
**Item** ^*^	**Disagree (n, %)**	**Agree (n, %)**
**Attitude section**		
Keeping track of the daily COVID-19 cases is important for the society	25 (6.10%)	385 (93.90%)
After knowing the number of COVID-19 cases, I feel worried/scared	154 (37.56%)	256 (62.44%)
Keeping track of governmental regulations to prevent the spread of COVID-19 is important for the society	25 (6.10%)	385 (93.90%)
All COVID-19 infected patients are people who disobey the public health regulations that are enforced to prevent the spread of the virus	182 (44.39%)	228 (55.61%)
People infected with COVID-19 should not be given a negative stigma by the society	43 (10.49%)	367 (89.51%)
COVID-19 patients who self-isolate themselves show a good responsibility to prevent the COVID-19 transmission	32 (7.80%)	378 (92.20%)

* Translated to English language.

**Table 2:** Frequencies and percentages of every response in each item in practice section.

Item^*^	**Never (*n, %*)**	**Rarely (*n, %*)**	**Occasionally (*n, %*)**	**Always (*n, %*)**
I wash my hands with soap or use hand sanitizer after touching surfaces in public	3 (0.73%)	6 (1.46%)	67 (16.34%)	334 (81.46%)
I take a bath and change my clothes every time I arrive home	2 (0.49%)	22 (5.37%)	136 (33.17%)	250 (60.98%)
I use mask in public areas (market, terminals, mosque, church, etc.)	1 (0.24%)	3 (0.73%)	15 (3.66%)	391 (95.37%)
I maintain at least a meter from other people when in public	5 (1.22%)	9 (2.20%)	79 (19.27%)	317 (77.32%)
I maintain my distance with elderly people	32 (7.80%)	49 (11.95%)	150 (36.59%)	179 (43.66%)
I attend events that have more than 50 people	130 (31.71%)	88 (21.46%)	101 (24.63%)	91 (22.20%)
I use public facilities or go to public places (public transportation, malls, markets, and tourist attraction)	136 (33.17%)	127 (30.98%)	87 (21.21%)	60 (14.63%)
I eat vegetables and fruits	5 (1.22%)	13 (3.17%)	104 (25.37%)	288 (70.24%)
I regularly do physical activities/sports	16 (3.90%)	78 (19.02%)	202 (49.27%)	114 (27.80%)
I have enough rest/sleep everyday	1 (0.24%)	28 (6.83%)	117 (28.54%)	264 (64.39%)
I consume vitamin or supplements to increase my immune system	37 (9.02%)	62 (15.12%)	131 (31.95%)	180 (43.90%)
Since the COVID-19 pandemic, I buy cleaning tools or products for self-hygiene, such as soap, hand sanitizer, etc.	9 (2.20%)	30 (7.32%)	79 (19.27%)	292 (71.22%)
I regularly clean my house/room.	1 (0.24%)	4 (0.98%)	39 (9.51%)	366 (89.27%)

* Translated to English language.

### Validity and reliability of instrument

Prior starting the study with the researcher-made questionnaire, 99 participants were recruited randomly to test for its validity and reliability. Verbal and written inform consent were required before individuals completed the questionnaire. To test for validity of each section (knowledge, attitude, and practice), Pearson's correlation between each item to the total score of the section was employed. All 22 items in the knowledge section, six6 items in the attitude section and 13 items in the practice section had significant correlation (*p* < 0.05) with the total score of the corresponding section. This indicated an acceptable validity for every item in the questionnaire.

Chronbach's Alpha coefficient was calculated to assess for reliability. The coefficients of knowledge, attitude and practice section of the survey were 0.75, 0.82, and 0.74, respectively, showing justifiable consistency (16).

### Statistical analysis

Descriptive and inferential statistics were conducted with the use of IBM SPSS version 22.0 (IBM Corp., Armonk, NY, USA). Continuous variables were presented using mean and standard deviation (SD), while categorical variables employing frequency and percentage. Chi-square test was performed to determine any association between each of the sociodemographic variables (age group, gender, education, occupation, and regency of residence) and each of the KAP variables. Age was grouped according to the classification set by WHO [young-age (18–43 years old); middle-age (44–59 years old), and old-age (60–74 years old)] (17). Bivariate correlation analysis was also conducted to assess the strength and direction of relationships of knowledge-attitude, knowledge-practice, and attitude-practice scores. Person's correlation was employed if both variables were normally distributed, otherwise Spearman's correlation was used. Normality of data distribution was evaluated by histograms, Quantile-Quantile (Q-Q) plot and Shapiro-Wilk test. The odds ratio was also calculated in every category. Last, linear regression analysis was done using socio-demographic variables, knowledge and attitude scores as the independent variables and practice score as the dependent variable, with the aim to explore factors associated with COVID-19 related practice. Statistically significant was defined when *p-*value was less than 0.05.

## Results

### Characteristics of respondents

Out of 434 participants who filled in the survey, only 410 people were included in analysis. Two people were excluded due to not residing permanently in NTT, one person submitted twice online and the remainder had incomplete information. The mean age of all participants was 32.70 ± 11.20 years (range 18–73). The majority of respondents (71.2%) was female (*n* = 292). More than three-quarter of participants lived in Kupang City (43.7%) and Kupang Regency (32.0%). Only four individuals (1%) indicated prior COVID-19 infection and 23 people (5.6%) had comorbidities as follows: hypertension (*n* = 13), diabetes mellitus type 2 (*n* = 3), epilepsy (*n* = 1), prostatic carcinoma (*n* = 1), asthma (*n* = 2), heart disease (*n* = 2), and major depressive disorder (*n* = 1). The elaborate frequencies and percentages in each of the socio-demographic variables were comprehensively outlined in Table 3.

**Table 3:** Knowledge toward COVID-19 by socio-demographic characteristics (*n* = 410).

**Variables**	**Frequency (%)**	**Good (*n, %*)**	**Bad (*n, %*)**	*P*-value^*^	**Cramer's V**
**Age group**				0.95	
Young	339 (82.68%)	271 (79.94%)	68 (20.06%)	
Middle	60 (14.63%)	47 (78.33%)	13 (21.67%)	
Elderly	11 (2.83%)	9 (81.81%)	2 (18.18%)	
**Sex**				0.76	
Male	118 (28.78%)	93 (78.81%)	25 (21.19%)	
Female	292 (71.22%)	234 (80.14%)	58 (19.86%)	
**Regency**				0.01	0.27
Kupang City	179 (43.66%)	147 (82.12%)	32 (17.88%)	
Alor	2 (0.49%)	1 (50.00%)	1 (50.00%)	
Belu	2 (0.49%)	1 (50.00%)	1 (50.00%)	
Ende	3 (0.73%)	3 (100.00%)	0 (50.00%)	
East Flores	13 (3.17%)	13 (100.00%)	0 (0%)	
Kupang Regency	131 (31.95%)	94 (71.76%)	37 (28.24%)	
Lembata	3 (0.73%)	3 (100.00%)	0 (0%)	
Malaka	4 (0.98%)	4 (100.00%)	0 (0%)	
Manggarai	2 (0.49%)	1 (50.00%)	1 (50.00%)	
West Manggarai	7 (1.71%)	6 (85.71%)	1 (14.29%)	
East Manggarai	2 (0.49%)	2 (100.00%)	0 (0%)	
Nagekeo	2 (0.49%)	2 (100.00%)	0 (0%)	
Ngada	3 (0.73%)	3 (100.00%)	0 (0%)	
Rote Ndao	19 (4.63%)	12 (63.16%)	7 (36.84%)	
Sabu Raijua	2 (0.49%)	2 (100.00%)	0 (0%)	
Sikka	9 (2.20%)	9 (100.00%)	0 (0%)	
West Sumba	2 (0.49%)	2 (100.00%)	0 (0%)	
Southwest Sumba	4 (0.98%)	3 (75.00%)	1 (25.00%)	
Central Sumba	2 (0.49%)	1 (50.00%)	1 (50.00%)	
East Sumba	10 (2.44%)	10 (100.00%)	0 (0%)	
South Central Timor	3 (0.73%)	2 (66.67%)	1 (33.33%)	
North Central Timor	6 (1.46%)	6 (100.00%)	0 (0%)	
**Occupation**				< 0.001	0.31
Entrepreneur	29 (7.07%)	20 (68.97%)	9 (31.03%)	
Police and national armed force	4 (0.98%)	2 (50.00%)	2 (50.00%)	
Unemployed	114 (27.80%)	80 (70.18%)	34 (29.82%)	
Student	39 (9.51%)	36 (92.31%)	3 (7.69%)	
Healthcare workers	47 (11.46%)	45 (95.74%)	2 (4.26%)	
Unskilled labor	53 (12.93%)	33 (62.26%)	20 (37.74%)	
Civil servant	52 (12.68%)	48 (92.31%)	4 (7.69%)	
Private employee	47 (11.46%)	42 (89.36%)	5 (10.64%)	
Teacher	25 (6.10%)	21 (84.00%)	4 (16.00%)	
**Education**				< 0.001	0.31
No formal education	2 (0.49%)	2 (100.00%)	0 (0%)	
Primary education	32 (7.80%)	15 (46.88%)	17 (53.13%)	
Secondary education	178 (43.41%)	131 (73.60%)	47 (26.40%)	
Higher education	198 (48.29%)	179 (90.40%)	19 (9.60%)	

* P-value for chi-square test to find any significant association between each of the variables with knowledge.

### COVID-19 knowledge

The interpretation of Cramer's V value was >0.25 = very strong association, >0.15 = strong association, >0.10 = moderate association, >0.05 = weak association, >0 = no or very weak association (18). The mean score for knowledge in this study was 17.91 ± 2.95 (range 5–22). Thus, the general knowledge score rate was at 81.4%. Overall, 327 (79.8%) respondents had good knowledge, while the remaining (20.2%) showed bad knowledge. The proportion of respondents having good and bad knowledge based on socio-demographic characteristics was outlined in Table 3. This study found statistically significant strong associations between knowledge and each of these following variables: respondents' regency of residence (Cramer's V = 0.266; *p* = 0.010), education (Cramer's V = 0.312; *p* < 0.001), and occupation (Cramer's V = 0.313; *p* < 0.001). This study also found significantly lower odds to have bad knowledge in students, healthcare workers, civil servant, and private employee compared to unemployed. Other than that, respondents with primary and secondary education background have higher odds to have bad knowledge compared to higher education.

### COVID-19 attitude

This study demonstrated a mean score of 4.88 ± 1.27 (range 0–6) for attitude toward COVID-19. Hence, the general attitude score rate was at 81.3%. Generally, 298 (72.7%) respondents had good attitude, whereas 112 (27.3%) had bad attitude. Table 4 presented the frequencies and percentages of both attitudes based on each of the socio-demographic variables. This study showed a strong association only between attitude and the respondents' regency of residence, and it was statistically significant (Cramer's V = 0.289; *p* = 0.024).

**Table 4:** Attitude toward COVID-19 by socio-demographic characteristics (*n* = 410).

**Variables**	**Frequency (%)**	**Good (*n, %*)**	**Bad (*n, %*)**	*P*-value^*^	**Cramer's V**
**Age group**				0.27	
Young	339 (82.68%)	241 (71.09%)	98 (28.91%)	
Middle	60 (14.63%)	48 (80.00%)	12 (20.00%)	
Elderly	11 (2.83%)	9 (81.81%)	2 (18.18%)	
**Sex**				0.30	
Male	118 (28.78%)	90 (76.27%)	28 (23.73%)	
Female	292 (71.22%)	208 (71.23%)	84 (28.77%)	
**Regency**				0.02	0.29
Kupang City	179 (43.66%)	138 (77.09%)	41 (22.91%)	
Alor	2 (0.49%)	2 (100.00%)	0 (0%)	
Belu	2 (0.49%)	2 (100.00%)	0 (0%)	
Ende	3 (0.73%)	1 (33.33%)	2 (66.67%)	
East Flores	13 (3.17%)	9 (69.23%)	4 (30.77%)	
Kupang Regency	131 (31.95%)	96 (73.28%)	35 (26.72%)	
Lembata	3 (0.73%)	2 (66.67%)	1 (33.33%)	
Malaka	4 (0.98%)	4 (100.00%)	0 (0%)	
Manggarai	2 (0.49%)	1 (50.00%)	1 (50.00%)	
West Manggarai	7 (1.71%)	1 (14.29%)	6 (85.71%)	
East Manggarai	2 (0.49%)	1 (50.00%)	1 (50.00%)	
Nagekeo	2 (0.49%)	2 (100.00%)	0 (0%)	
Ngada	3 (0.73%)	2 (66.67%)	1 (33.33%)	
Rote Ndao	19 (4.63%)	12 (63.16%)	7 (36.84%)	
Sabu Raijua	2 (0.49%)	2 (100.00%)	0 (0%)	
Sikka	9 (2.20%)	8 (88.89%)	1 (11.11%)	
West Sumba	2 (0.49%)	0 (0%)	2 (100.00%)	
Southwest Sumba	4 (0.98%)	2 (50.00%)	2 (50.00%)	
Central Sumba	2 (0.49%)	2 (100.00%)	0 (0%)	
East Sumba	10 (2.44%)	6 (60.00%)	4 (40.00%)	
South Central Timor	3 (0.73%)	1 (33.33%)	2 (66.67%)	
North Central Timor	6 (1.46%)	4 (66.67%)	2 (33.33%)	
**Occupation**				0.19	
Entrepreneur	29 (7.07%)	21 (72.41%)	8 (27.59%)	
Police and national armed force	4 (0.98%)	3 (75.00%)	1 (25.00%)	
Unemployed	114 (27.80%)	85 (74.56%)	29 (25.44%)	
Student	39 (9.51%)	30 (76.92%)	9 (23.08%)	
Healthcare workers	47 (11.46%)	41 (87.23%)	6 (12.77%)	
Unskilled labor	53 (12.93%)	39 (73.58%)	14 (26.42%)	
Civil servant	52 (12.68%)	33 (63.46%)	19 (36.54%)	
Private employee	47 (11.46%)	30 (63.83%)	17 (36.17%)	
Teacher	25 (6.10%)	16 (64.00%)	9 (36.00%)	
**Education**				0.29	
No formal education	2 (0.49%)	1 (50.00%)	1 (50.00%)	
Primary education	32 (7.80%)	19 (59.38%)	13 (40.63%)	
Secondary education	178 (43.41%)	129 (72.47%)	49 (27.53%)	
Higher education	198 (48.29%)	149 (75.25%)	49 (24.74%)	

* P-value for chi-square test to find any significant association between each of the variables with attitude.

### COVID-19 practice

The mean score for practice was 44.42 ± 4.39 (range 19–52). Therefore, the general practice score rate was at 85.4%. A total of 388 (94.6%) respondents exhibited a good COVID-19 related practice, while the rest (5.4%) showed otherwise. Table 5 illustrated the proportion of good and bad practice based on each of the socio-demographic variables. This study showed a strong association that was statistically significant between practice and the respondents' education (Cramer's V = 0.272; *p* < 0.001). Table 6 summarized the odds of obtaining poor knowledge, attitude, and practice based on each socio-demographic variable. This study also showed that respondents from rural area, respondents with a primary and secondary education background, and unskilled labors have higher odds to show poor COVID-19 practice.

**Table 5:** Practice toward COVID-19 by socio-demographic characteristics (*n* = 410).

**Variables**	**Frequency (%)**	**Good (*n, %*)**	**Bad (*n, %*)**	*P*-value^*^	**Cramer's V**
**Age group**				0.38	
Young	339 (82.68%)	319 (94.10%)	20 (5.90%)	
Middle	60 (14.63%)	58 (96.67%)	2 (3.33%)	
Elderly	11 (2.83%)	11 (100.00%)	0 (0%)	
**Sex**				0.08	
Male	118 (28.78%)	108 (91.53%)	10 (8.47%)	
Female	292 (71.22%)	280 (95.89%)	12 (4.11%)	
**Regency**				0.57	
Kupang City	179 (43.66%)	175 (97.77%)	4 (2.23%)	
Alor	2 (0.49%)	2 (100.00%)	0 (0%)	
Belu	2 (0.49%)	2 (100.00%)	0 (0%)	
Ende	3 (0.73%)	3 (100.00%)	0 (0%)	
East Flores	13 (3.17%)	12 (92.31%)	1 (7.69%)	
Kupang Regency	131 (31.95%)	122 (93.13%)	9 (6.87%)	
Lembata	3 (0.73%)	3 (100.00%)	0 (0%)	
Malaka	4 (0.98%)	4 (100.00%)	0 (0%)	
Manggarai	2 (0.49%)	1 (50.00%)	1 (50%)	
West Manggarai	7 (1.71%)	6 (85.71%)	1 (14.29%)	
East Manggarai	2 (0.49%)	2 (100.00%)	0 (0%)	
Nagekeo	2 (0.49%)	2 (100.00%)	0 (0%)	
Ngada	3 (0.73%)	3 (100.00%)	0 (0%)	
Rote Ndao	19 (4.63%)	16 (84.21%)	3 (15.79%)	
Sabu Raijua	2 (0.49%)	2(100.0%)	0 (0%)	
Sikka	9 (2.20%)	8 (88.89%)	1 (11.11%)	
West Sumba	2 (0.49%)	2 (100.00%)	0 (0%)	
Southwest Sumba	4 (0.98%)	3 (75.00%)	1 (25.00%)	
Central Sumba	2 (0.49%)	2 (100.00%)	0 (0%)	
East Sumba	10 (2.44%)	10 (100.00%)	0 (0%)	
South Central Timor	3 (0.73%)	3 (100.00%)	0 (0%)	
North Central Timor	6 (1.46%)	5 (83.33%)	1 (16.67%)	
Occupation				0.07	
Entrepreneur	29 (7.07%)	27 (93.10%)	2 (6.90%)	
Police and national armed force	4 (0.98%)	4 (100.00%)	0 (0%)	
Unemployed	114 (27.80%)	108 (94.74%)	6 (5.26%)	
Student	39 (9.51%)	37 (94.87%)	2 (5.13%)	
Healthcare workers	47 (11.46%)	46 (97.87%)	1 (2.13%)	
Unskilled labor	53 (12.93%)	45 (84.91%)	8 (15.09%)	
Civil Servant	52 (12.68%)	52 (100.00%)	0 (0%)	
Private Employee	47 (11.46%)	45 (95.74%)	2 (4.26%)	
Teacher	25 (6.10%)	24 (96.00%)	1 (4.00%)	
**Education**				< 0.001	0.27
No formal education	2 (0.49%)	2 (100.00%)	0 (0%)	
Primary education	32 (7.80%)	24 (75.00%)	8 (25.00%)	
Secondary education	178 (43.41%)	167 (93.82%)	11 (6.18%)	
Higher education	198 (48.29%)	195 (98.48%)	3 (1.52%)	

* P-value for chi-square test to find any significant association between each of the variables with practice.

**Table 6:** Odds ratio and 95% confidence interval (CI) for odds ratio of the logistic regression for poor knowledge, poor attitude and poor practice (*n* = 410).

**Variables**	**Knowledge**		**Attitude**		**Practice**	
	*P*-value^*^	**OR (95% CI)**	*P*-value^*^	**OR (95% CI)**	*P*-value^*^	**OR (95% CI)**
**Age group**					
Young^¶^	**Reference for OR**					
Middle	0.775	1.102 (0.564–2.153)	0.158	0.615 (0.313–1.207)	0.429	0.550 (0.125–2.417)
Elderly	0.878	0.886 (0.186–4.194)	0.445	0.546 (0.116–2.575)	0.999	0.000
**Sex**						
Male^¶^	**Reference for OR**					
Female	0.763	0.922 (0.544–1.562)	0.301	1.298 (0.792–2.127)	0.082	0.463 (0.194–1.103)
**Regency**						
Urban areas^¶^	**Reference for OR**					
Rural areas	0.295	1.302 (0.795–2.130)	0.078	1.494 (0.955–2.335)	0.020	3.697 (1.229–11.126)
**Occupation**						
Entrepreneur	0.899	1.059 (4.38–2.561)	0.814	1.117 (0.446–2.793)	0.733	1.333 (0.255–6.977)
Police and national armed force	0.402	2.353 (0.318–17.397)	0.984	0.977 (0.098–9.765)	0.999	0.000
Unemployed^¶^	**Reference for OR**					
Student	0.010	0.196 (0.057–0.680)	0.768	0.879 (0.374–2.069)	0.974	0.973 (0.188–5.032)
Healthcare workers	0.003	0.105 (0.024–0.456)	0.082	0.429 (0.165–1.114)	0.391	0.391 (0.046–3.342)
Unskilled labor	0.310	1.426 (0.719–2.829)	0.893	1.052 (0.501–2.210)	0.041	3.2 (1.050–9.750)
Civil servant	0.004	0.196 (0.066–0.587)	0.145	1.688 (0.834–3.413)	0.997	0.000
Private employee	0.014	0.280 (0.102–0.769)	0.173	1.661 (0.801–3.444)	0.789	0.800 (0.156–4.114)
Teacher	0.168	0.448 (0.143–1.404)	0.286	1.649 (0.658–4.133)	0.794	0.750 (0.086–6.521)
**Education**						
No formal education	0.999	0.000	0.435	3.041 (0.187–49.534)	1	0
Primary education	< 0.001	10.677 (4.609–24.734)	0.064	2.081 (0.958–4.520)	< 0.001	21.667 (5.381–87.249)
Secondary education	< 0.001	3.380 (1.895–6.028)	0.540	1.155 (0.729–1.831)	0.028	4.281 (1.175–15.604)
Higher education^¶^	**Reference for OR**					

* P-value for odds ratio.

¶ Reference variable is young, male, urban areas, unemployed, and higher education. OD, odds ratio.

### Correlation between KAP variables

This study used the correlations criteria of the following: r or ρ(rho) value 0–0.3 = poor correlation, 0.3–0.6 = fair correlation, 0.6–0.8 = moderate correlation, and 0.8–1 = very strong correlation (18). The scores of knowledge, attitude, and practice were not normally distributed based on the histogram, Q-Q plot, and Shapiro-Wilk test (*p* < 0.001). This study indicated no significant correlation between knowledge and attitude scores (*p* = 0.721), and also between attitude and practice scores (*p* = 0.160). However, there was a poor positive linear correlation that was significant between knowledge and practice scores (ρ = 0.097; *p* = 0.049).

A multiple regression analysis was conducted to predict practice score from sex, age, occupation, education, knowledge and attitude scores. These variables significantly predicted practice score, *F*_(15, 394)_ = 5.700 (*p* < 0.001), *R*^2^ = 0.178. Only age (*B* = 0.059), being male (*B* = −1.434), having unskilled labor occupation (*B* = −1.457), and attitude scores (*B* = 0.328) were significantly associated with lower practice scores (*p* < 0.05).

To test our hypothesis, data collected from the questionnaires were analyzed using chi-square to determine the association between the sociodemographic variables and KAP. Pearson's correlation was also chosen to assess the strength and direction of the relationship between COVID-19 knowledge, attitude and practice. Linear regression analysis was done to predict the practice score as a dependent variable based on the sociodemographic variables.

## Discussion

In this study with predominantly young, female population, nearly eighty-percent of the NTT respondents displayed good knowledge on COVID-19 with an average score rate at 81.4% which was higher than studies conducted in China, Venezuela, Iran, United States and India (19–22). This relatively high score was possibly due to different public health actions taken by the respective government and also this study took place during the rapid rise of cases in which the local authorities enforced more COVID-19 campaigns and educations to raise awareness among the communities. Similar to previous studies, positive relationship of knowledge-practice scores was consistent with a social cognitive theory describing individual knowledge as a factor in determining one's behavior alongside with environmental factors (20, 23–25). Therefore, it is necessary for uniform distribution of reliable COVID-19 information throughout all communities from the local public health agency in order to promote behavioral change thereby reducing virus spread.

This study revealed a strong association between regency of residence and COVID-19 knowledge, which highlights the importance of accessibility of knowledge in all areas. Education and occupation were also a strong factor in the knowledge of COVID-19, which was aligned with other studies (19, 20, 23, 26). Students, healthcare workers, civil servant, and private employees have lower odds to have bad knowledge compared to unemployed. Healthcare workers were more likely to have good knowledge, which is also align with a study in Iran (26). Students were more likely to have better knowledge compared to unemployed as health campaigns are often displayed in schools, which further accentuates the importance of education as well. Civil servants were more likely to have better knowledge compared to unemployed as they may be more exposed to governmental policies and campaigns. Moreover, respondents with a background education of primary and secondary education have higher odds to have bad knowledge compared to higher education, which is aligned with the presumption that people who had a higher educational level will have better understanding and is aligned with other studies (19, 27–32). This discrepancy between occupations and education levels further solidify the need for personalized campaigns and better dissemination of information especially when the majority of Indonesia consists of primary education graduates and more than 10 million people are unemployed.

There were several knowledge gaps found in this study, indicating ineffective public education that has been done by the local health authorities. About half of the participants believed that the novel coronavirus could not penetrate fabric masks and people with robust immune system cannot be infected with the virus, which were both inaccurate. These misconceptions should be addressed to avoid false assumptions that having a strong immune system and wearing fabric masks without following additional precautionary behaviors like social distancing were adequate to not contract the virus.

Nearly three-quarter of the participants had good attitude toward COVID-19, conveying considerable proportion of people understood the seriousness of COVID-19 and urgency in executing precautionary measures set by the public health agency in order to downscale the transmission. This proportion was moderately higher than the result shown in Yanti et al. study among the general Indonesian population which was less than 60% (7), which difference was presumably due to this study was conducted over a year after the start of COVID-19 pandemic and expressed attitude of the population toward it may change over time as more people feared the uncontrollable surge of COVID-19 in addition to the collapse of Indonesia's healthcare system (33). However, other studies conducted in Indonesia and other countries showed higher attitude, possibly due to difference of target population where other studies cover the population more extensively (28, 34–36). Similar with studies done in Venezuela and Iran (20, 26), this survey found a statistically significant strong relationship between regency of residence and the attitude regarding COVID-19.

Vast majority (~95%) of participants showed good COVID-19 related practice. This higher percentage compared to the proportion of good knowledge and attitude could be due to the nationwide lockdown and sanction imposed by the Indonesian authorities for individuals violating COVID-19 regulations set by the national public health agency thereby forcing all residents to follow COVID-19 advices. This finding was in line with the research performed among all Indonesian population during initial phase of the pandemic (7). Education was a factor which significantly associated to COVID-19 related practice, and respondents with a background of primary and secondary education have higher odds to show bad practice compared to higher education group. This was consistent with studies in other third world countries like Northeast Ethiopia and Nepal (23, 37). Unlike other Indonesian provinces, majority of NTT population has either low or no education especially people living in remote areas. This raises a concern regarding many less-educated people presumably ignoring the importance of good COVID-19 practices and it may explain why NTT has one of the highest daily cases in Indonesia during the second wave of the pandemic (July–August 2021). This study showed that rural areas of NTT have higher odds to show bad practice compared to urban areas, which is aligned with the assumption that rural areas have thicker cultural beliefs thereby perceiving COVID-19 trivially (23, 38–40). This gap between the rural and urban areas were also seen in other studies. Thus, policy planners and public health authorities should give more attention to people with lower education and living in more rural areas by stressing that the threat caused by the pandemic existed, emphasizing on the severity and vulnerability, and providing proof of examples that the suggested advices will avert it (41). This focus is crucial as less educated people and rural areas are more exposed to a risk of high death rate due to difficulty of healthcare access.

Almost half of the participants stated that they still attended events with more than 50 people despite vast majority of them (~94%) understood well that traveling to crowded places could help COVID-19 transmission. The reason is likely to be various tight cultures and social obligations among NTT residents that require them to gather in events even though most people comprehend the risk. Social norms and culture are factors that could influence one's behavior and there are situations when these are prioritized more than security thereby making the pandemic difficult to be controlled (42). There was still sizeable proportion of people who still used public transportation or went to public places even though majority knew the risk. This is because of most people living in NTT has low paying blue-collar jobs which require them to travel to work every day in order to survive and provide for their families. Policy and decision makers could impose a tighter regulation on public facilities to resolve the issue like implementing social distancing and reducing operational hours. Another way is for the central government to provide economic relief or assistance especially for the poor communities. Identical with previous studies, age and male gender were significantly associated with lower practice scores (23, 24, 26).

There are several strength and limitations of this study. The samples in this research involved all 22 regencies of NTT, including areas that are far from health centers and rarely gets information from outside. Moreover, the questionnaire used had been tested for validity and reliability, which bolsters the validity of our findings. Although this study covered all regencies across NTT, most of the respondents were in the capital city. Therefore, there is lack of generalizability in this research. Other limitations of this study include the potential presence of social-desirability bias due to self-report data and selection bias due to non-probability sampling implemented in this study. The evolving nature of COVID-19 updates can also be a threat to our study's findings. The nature of this study, which is cross-sectional cannot determine direct causality between the variables. Further research is recommended to reassert and track changes, if any, of the KAP among NTT population especially after recommended advices have been taken place. Confounding variables can be further explored to further solidify the study's findings.

Satisfactory knowledge and attitude are crucial in a pandemic where the public is urged to perform appropriate practice to control the spread. However, the COVID-19 pandemic is a new and unpredictable situation where it needs personalized and up-to-date report regarding the status of the knowledge, attitude, and practice to contrive plans that are unique to a population and sociodemographic background. This study enforces the correlation between knowledge and practice and the association of various sociodemographic factors to KAP. However, this study also found that there were existing disparities of KAP across sociodemographic categories, therefore public health officials should take appropriate measures especially to vulnerable groups.

## Data availability statement

The original contributions presented in the study are included in the article/supplementary material, further inquiries can be directed to the corresponding author.

## Ethics statement

This study obtained ethical approval from Faculty of Public Health Universitas Airlangga – ERB Registration No. 47/EA/KEPK/2021. The patients/participants provided their written informed consent to participate in this study.

## Author contributions

FL developed conception and design of the study, conducted data acquisition, analysis, interpretation, drafted, revised the work, and subsequently approved the final version to be published. AS contributed in developing of study design, performed data analysis and interpretation, drafted, revised the work, and ultimately took part in final approval of the version to be submitted and published. Both authors contributed to the article and approved the submitted version.

## Conflict of interest

The authors declare that the research was conducted in the absence of any commercial or financial relationships that could be construed as a potential conflict of interest.

## Publisher's note

All claims expressed in this article are solely those of the authors and do not necessarily represent those of their affiliated organizations, or those of the publisher, the editors and the reviewers. Any product that may be evaluated in this article, or claim that may be made by its manufacturer, is not guaranteed or endorsed by the publisher.
